# Anti-HIV Activity in Cervical-Vaginal Secretions from HIV-Positive and -Negative Women Correlate with Innate Antimicrobial Levels and IgG Antibodies

**DOI:** 10.1371/journal.pone.0011366

**Published:** 2010-06-29

**Authors:** Mimi Ghosh, John V. Fahey, Zheng Shen, Timothy Lahey, Susan Cu-Uvin, Zhijin Wu, Kenneth Mayer, Peter F. Wright, John C. Kappes, Christina Ochsenbauer, Charles R. Wira

**Affiliations:** 1 Department of Physiology, Dartmouth Medical School, Lebanon, New Hampshire, United States of America; 2 Department of Microbiology and Immunology and Department of Medicine, Dartmouth Medical School, Lebanon, New Hampshire, United States of America; 3 The Immunology Center, The Miriam Hospital, Brown University, Providence, Rhode Island, United States of America; 4 Department of Community Health and Center for Statistical Sciences, Brown University, Providence, Rhode Island, United States of America; 5 Department of Pediatrics, Dartmouth Medical School, Lebanon, New Hampshire, United States of America; 6 Department of Medicine, University of Alabama, Birmingham, Alabama, United States of America; 7 Department of Microbiology, University of Alabama, Birmingham, Alabama, United States of America; 8 Department of Pathology, University of Alabama, Birmingham, Alabama, United States of America; Karolinska Institutet, Sweden

## Abstract

**Background:**

We investigated the impact of antimicrobials in cervicovaginal lavage (CVL) from HIV(+) and HIV(−) women on target cell infection with HIV. Since female reproductive tract (FRT) secretions contain a spectrum of antimicrobials, we hypothesized that CVL from healthy HIV(+) and (−) women inhibit HIV infection.

**Methodology/Principal Findings:**

CVL from 32 HIV(+) healthy women with high CD4 counts and 15 healthy HIV(−) women were collected by gently washing the cervicovaginal area with 10 ml of sterile normal saline. Following centrifugation, anti-HIV activity in CVL was determined by incubating CVL with HIV prior to addition to TZM-bl cells. Antimicrobials and anti-gp160 HIV IgG antibodies were measured by ELISA. When CXCR4 and CCR5 tropic HIV-1 were incubated with CVL from HIV(+) women prior to addition to TZM-bl cells, anti-HIV activity in CVL ranged from none to 100% inhibition depending on the viral strains used. CVL from HIV(−) controls showed comparable anti-HIV activity. Analysis of CH077.c (clone of an R5-tropic, mucosally-transmitted founder virus) viral inhibition by CVL was comparable to laboratory strains. Measurement of CVL for antimicrobials HBD2, trappin-2/elafin, SLPI and MIP3**α** indicated that each was present in CVL from HIV(+) and HIV(−) women. HBD2 and MIP3α correlated with anti-HIV activity as did anti-gp160 HIV IgG antibodies in CVL from HIV(+) women.

**Conclusions/Significance:**

These findings indicate that CVL from healthy HIV(+) and HIV(−) women contain innate and adaptive defense mechanisms that inhibit HIV infection. Our data suggest that innate endogenous antimicrobials and HIV-specific IgG in the FRT can act in concert to contribute toward the anti-HIV activity of the CVL and may play a role in inhibition of HIV transmission to women.

## Introduction

Heterosexual transmission of HIV is the predominant driver of the growing HIV pandemic [1], [2]. Yet, while considerable attention has been directed to developing topical exogenous microbicides that reduce transmission of HIV-1, relatively little is known about endogenous microbicides produced within the female reproductive tract (FRT). That endogenous microbicides in the female reproductive tract secretions might limit or prevent HIV transmission is suggested by the relatively low risk of HIV transmission per heterosexual coitus, from 1∶122 to 1∶1000 [3], [4].

We and others have shown that cells of the FRT produce and secrete a spectrum of cytokines, chemokines, and antimicrobials [5]–[8]. Several specifically inhibit HIV infection of target cells [9], [10]. Antimicrobials secreted by FRT cells include well-characterized anti-HIV molecules, alpha/beta defensins, lactoferrin, and secretory leukocyte protease inhibitor (SLPI), as well as factors such as trappin-2/elafin and MIP3**α**, which have recently been shown to have anti-HIV activity [9]–[13]. Some of these factors such as human beta defensins 2 (HBD2) act directly to inhibit virus [10], while others including SDF1α, RANTES, MIP1α, and MIP1β bind to co-receptors to prevent viral entry into target cells [14]. Recent studies have linked the presence of cationic polypeptides in CVL to anti-HSV and anti-HIV activity [15], [16]. Venkataraman *et al.* showed that when all cationic polypeptides were depleted from the CVL, antimicrobial activity was lost [16].

The isolation of HIV-1 in the FRT was first reported in 1986 [17]. Since then, numerous studies have reported the presence of cell-free HIV-1 RNA, cell-associated HIV-1 RNA, proviral DNA, and culturable virus from the cervix and vagina of pregnant and non-pregnant infected women [18]–[20]. While it is clear that HIV-1 is shed into the FRT, a detailed understanding of this phenomenon and factors that affect the amount and infectivity of virus in the FRT has not yet been elucidated. Reichelderfer *et al.* reported that HIV-1 RNA levels in endocervical secretions were highest in the week preceding menses [21]. Other studies have shown no effect of the menstrual cycle on the amount or infectivity of HIV-1 in the FRT [22]. In a recent study, the number of HIV-1 infected cells in endocervical secretions was reported to increase at midcycle just after the periovulatory phase [23]. In other studies, Cummins and colleagues showed that certain innate immune factors in vaginal lavages were more closely associated with HIV-1 shedding in the genital mucosa than plasma viral load [24]. Whether virus is shed into the vagina from the upper FRT remains to be determined. Whereas HIV-1 shedding in CVL secretions is readily detectable, it remains unclear what percentage of the shed virus is actually infectious [24], [25].

In this study, we assessed the levels of multiple candidate endogenous microbicides in cervicovaginal lavage (CVL) specimens from HIV(+) and HIV(−) women, and characterized whether these microbicides correlate with protection from HIV infection. Of the four microbicides analyzed, we found that the levels of two endogenous microbicides, HBD2 and MIP3α correlated with activity against HIV. Analysis of CVL for HIV-specific IgG of healthy HIV(+) women further indicated a positive correlation with anti-HIV activity. These data indicate that CVL from HIV(+) and HIV(−) women contain endogenously produced antimicrobials and IgG (HIV+ samples) that correlate with protection against HIV infection. Further, these findings suggest that, as a consequence of antimicrobial activity in the lower FRT, an environment exists for viral inactivation, which in part may contribute to the low frequency of infectious virus found in FRT secretions. The identification of endogenous microbicides that inhibit HIV transmission should contribute both to our understanding of the pathogenesis of HIV-1, as well as facilitate the development of novel microbicides capitalizing on existing host immune mechanisms.

## Methods

### HIV(+) Participants

Specimen collection and patient information were provided as a part of an observational study on HIV shedding in women. This study was conducted according to the principles expressed in the Declaration of Helsinki. The study was approved by the Miriam Hospital Institutional Review Board (Brown University, Providence, RI). All patients (HIV(+) and HIV(−)) provided written informed consent for the collection of samples and subsequent analysis. Thirty two HIV(+), sexually abstinent women (48 hrs prior to sampling) between the ages of 16 and 41 years were recruited from The Immunology Center, Miriam Hospital (Table 1). Enrollment criteria included a normal menstrual history, not on hormonal contraceptives, CD4 T cell counts above 350 cells/mm^3^ (mean 713 (419–1517) cells/mm3), and no exposure to antiretroviral (ARV) drugs. Participants agreed to undergo colposcopic assessment, and were excluded for pregnancy, breastfeeding, menopause, or inter-menstrual bleeding in the previous three months. Additionally, women were excluded if they had douched, used any vaginal products, or had sexual intercourse during the 48 hrs prior to CVL collection. CVL was collected by gently washing the cervicovaginal area with 10 ml of sterile normal saline (pH∼7.2). Following CVL collection, samples were centrifuged at 10,000×g for 5 min after which supernatants and cell pellets were stored at −80°C until used. Women were tested for lower genital tract infections including but not limited to bacterial vaginosis (BV), *Trichomonas vaginalis*, *Neisseria gonorrhea* and *Candida albicans*. Race and ethnicity were self-defined by the women involved in the study. Analysis of plasma viral load (PVL) and genital tract viral load (GTVL) RNA as well as CD4 counts, age and race are shown in Table 1.

**Table 1:** HIV-1 Patient Demographics^a^.

PATIENT SUBJECT #	RACE	AGE	PVL	GTVL	CD4 count
1*	B	33	<400	<400	419
2	B	23	23,000	<400	459
3	B	35	3,300	<400	510
4	B	41	12,000	<400	517
5	B	38	20,000	9700	580
6	B	27	32,000	<400	583
7	B	39	67,000	<400	628
8	B	30	1,500	<400	637
9*	B	31	<400	<400	647
10	B	33	17,000	<400	685
11*	B	27	6,629	<400	772
12	B	24	10,000	<400	863
13	H	31	840	<400	426
14	H	27	9,300	<400	510
15	H	36	<400	<400	620
16	H	28	<400	<400	799
17	H	32	54,000	<400	813
18	H	31	<400	<400	899
19	H	27	3,600	460,000	901
20	H	16	5,200	4,700	1,517
21	W	28	3,500	<400	496
22*	W	22	2,100	<400	502
23	W	29	110,000	300,000	605
24	W	26	3,400	<400	631
25*	W	18	8,000	<400	637
26	W	32	<400	<400	651
27	W	30	<400	<400	677
28	W	22	7,600	<400	822
29*	W	29	870	38,000	872
30*	W	19	<400	<400	921
31	W	25	<400	<400	931
32	W	32	1,800	<400	1,277

a CVL were recovered from HIV(+) women and stored in the HERS repository. The lower level of detection of our viral load assay was 400 copies/ml. Women self- identified by race as Black (B), Hispanic (H) or White (W). Also indicated is the age of each individual, plasma viral RNA load (PVL), genital tract viral RNA load (GTVL) and CD4 counts (cells/mm^3^).

*Denotes women with STDs; #1, 22, 25 had BV; #11 had Trichomonas; #9, 30 had BV/Trich; #29 had BV/fungus.

### HIV(−) Participants

CVL from participants in this study were obtained from the Rhode Island site (Miriam Hospital, Brown University, Providence, RI) of the HIV Epidemiology Research (HER) study. Women ranged in age from 24 to 34 yrs. Race was self-defined by the women involved in the study. CVL was collected by gently washing the cervicovaginal area with 10 ml of sterile normal saline (pH∼7.2). Following CVL collection, samples were immediately frozen at −80°C. At the time of analysis, samples were thawed to room temperature, centrifuged at 10,000×g for 5 min after which supernatants were assayed for anti-HIV activity. In preliminary studies (not shown), we compared CVL that were centrifuged after collection and prior to freezing with those that were freeze-thawed once prior to centrifugation and found no differences in anti-HIV inhibitory activity.

### HIV Viral Stocks

Laboratory-adapted viral strains HIV-1 IIIB (X4) and BaL (R5) were obtained from Dr. P. Gupta (Univ. of Pittsburgh, PA). Virus stocks were propagated in PHA-stimulated human PBMC and stored frozen at −80°C. Virus stocks produced in PBMC of molecularly cloned HIV-1 NL4.3 (X4; lab-adapted) and YU-2.c (R5; directly cloned without culture) were used in this study. Virus titers were determined on TZM-bl cells as described [26]. Also used was a PBMC-derived virus stock of pCH077.c, a recently generated Clade B infectious molecular clone ([27], [28], Ochsenbauer *et al.*, manuscript in preparation) matching the nucleotide sequence determined to be the transmitted/founder virus sequence of CHAVI subject 700010077.

### Measurement of infectious HIV in CVL

Intrinsic anti-HIV activity in CVL was determined using TZM-bl cells [26]. The TZM-bl indicator cell line is a HeLa cell derivative that expresses high levels of CD4, CCR5 and CXCR4. Cells contain HIV long terminal repeat (LTR)-driven β-galactosidase and firefly luciferase reporter cassettes that are activated by HIV infection and subsequent Tat protein expression [26]. TZM-bl cells were routinely subcultured every 3 to 4 days by trypsinization and maintained in TZM-bl media consisting of phenol red-free DMEM (Invitrogen Life Technologies, Carlsbad, CA) supplemented with 10% defined FBS (HyClone, Logan, UT, USA), 2 mM L-glutamine (Invitrogen Life Technologies), and 50 µg/ml primocin (Invivogen, San Diego, CA, USA).

To measure the presence of infectious HIV-1 in CVL, TZM-bl cells were seeded at 2×10^4^ cells per well in a 96-well microtiter plate and allowed to adhere overnight at 37°C. Patient CVL samples were diluted 1∶4 in TZM-bl media prior to 100µl being added to TZM-bl cells for 48h at 37°C in 5% CO_2_. Luciferase activity was measured following manufacturer's instructions. Briefly, following aspiration of supernatants, a beta-glo luciferase substrate (Promega, Madison, WI) solution (100µl) was added to cells. The light intensity of each well was measured using a luminometer. Background luminescence was determined by analyzing uninfected TZM-bl cells. All infectivity assays were performed in quadruplicate. Media diluted CVL had neutral pH values (pH 7.0–7.2).

### Measurement of CVL anti-HIV-1 activity

TZM-bl cells were seeded at 2×10^4^ cells per well in a 96-well microtiter plate and allowed to adhere overnight at 37°C. CVL from individual patients were diluted 1∶4 and incubated with virus (MOI = 1) for 1hr at 37°C in a final volume of 100µl. Following incubation, media was aspirated from TZM-bl cells and the virus plus CVL mixture (100µl) was added to the cells along with 100µl of TZM-bl media. Luciferase activity was measured as described above. Controls included incubation of TZM-bl with virus alone, CVL alone and cells in media. Uninfected cells were used to determine background luminescence and data was expressed in relative light units (RLU). To calculate percent inhibition, the RLU values of “virus only” wells were averaged and set to 100%. Values of CVL treated virus were calculated as a percentage of the “virus only” and then subtracted from 100 to calculate % inhibition. Viability of TZM-bl cells upon treatment with CVL was quantified using the CellTiter 96® AQueous One Solution Cell Proliferation Assay (Promega) according to manufacturer's instructions. Briefly, reagent was added directly to cell cultures and incubated for 30 min at 37°C followed by reading the plate in a plate reader at OD 490nm.

### Measurement of antimicrobials in CVL

CVL supernatants were stored at −80°C until assayed for SLPI, MIP3**α** and trappin-2/elafin with ELISA test kits or ELISA Duoset kit from R&D Systems (Minneapolis, MN) according to the manufacturer's protocol. Standards for each ELISA were re-suspended in phosphate buffered saline (PBS). Samples were also diluted in 1xPBS. Cytokines were quantified based on standard curves obtained using an ELISA reader (Dynex, Chantilly, VA). HBD2 was assayed with an ELISA test kit from PeproTech (Rocky Hill NJ) according to the manufacturer's protocol.

### Measurement of anti-gp160HIV IgA and IgG antibody levels in CVL

Specimens were tested by kinetic ELISA (kELISA) adapted from an assay previously described for influenza [29]. The kELISA measures substrate activation every 9 sec over the first 5 min of the enzymatic assay and plots the change in color per min as mOD/min using a Thermomax microplate reader (Molecular Devices, Sunnyvale, CA). Duplicate wells were coated with MN strain gp160 (Protein Sciences, Meriden, CT). An uncoated well was run with each sample to determine background, which was subtracted from the result. A standard curve with a known positive serum specimen was included in each assay as a measure of sensitivity and reproducibility. Biotinylated anti-human IgG and IgA conjugates were used with streptavidin-HRP and ABTS [29]. The presence of anti-HIV gp160 specific IgG and IgA antibodies used 15mOD as a cut-off for detection.

### Measurement of HIV-1 RNA in CVL and Plasma

Nucleic acid sequence-based amplification (BioMerieux, Durham, NC) was used to measure HIV-1 RNA as previously described [30]. All results are expressed as copies per ml with a lower limit of detection of 2.6 log_10_ (400) copies/ml for both plasma and CVL.

### Statistical analysis

Data were analyzed using GraphPad Prism (La Jolla, CA) and STATA 9 (College Station, TX). Mean and standard error were calculated for all data sets. Comparisons of anti-HIV activity between control and CVL groups were done using Mann-Whitney U tests, with a P value of less than 0.05 considered to be significant. We analyzed the correlation between CVL microbicide levels and the percent reduction in HIV infection of TZM-bl cells using Spearman correlation coefficients with a P value threshold for statistical significance of 0.05. The percent reduction in HIV infection of TZM-bl cells for each isolate was divided into quartiles, and a score assigned to each quartile. Then, to assess the impact of a given CVL microbicide with inhibition of target cell infection across viral strains, we assessed the correlation of levels of each endogenous microbicide with summed quartile scores using Spearman correlation coefficients as above.

## Results

### Infectious HIV-1 in Cervical Vaginal Lavage (CVL) specimens from HIV(+) women

**Figure 1:**
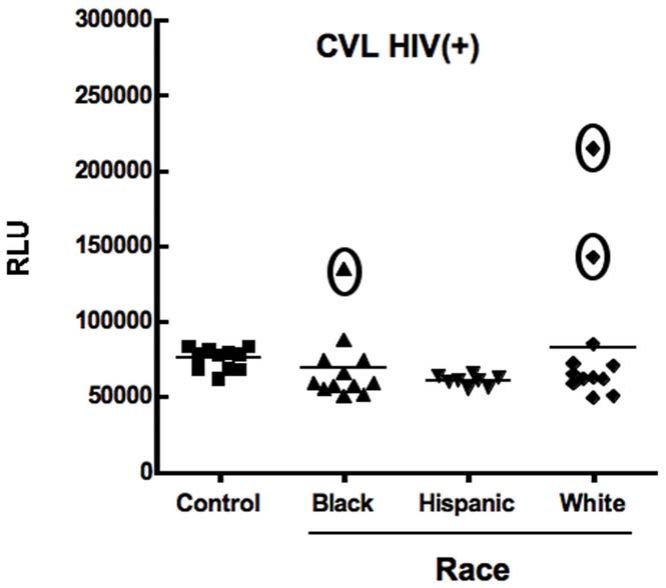
Determination of the presence of infectious HIV-1 in CVL from HIV(+) women. HIV(+) CVL from 32 patients were diluted 1∶4 and added directly to TZM-bl cells. The assay was terminated 48h post-infection and HIV-1 infection was quantified by measuring luciferase reporter gene activity using a luminometer and expressed as relative light units (RLU). Each data point in graph represents one individual patient. In the Control (media only) column, each point represents replicate wells. When comparing Controls with the CVL samples (TZM-bl cells incubated with CVL), 3 out of 32 showed RLU levels at least 2-fold above background indicating that these 3 CVL contained infectious HIV-1 which trans-activated the LTR-driven reporter gene.

Table 1 shows the patient profiles of HIV(+) women indicating age, race/ethnicity, CD4 count, plasma HIV viral load (PVL) and genital tract HIV viral load (GTVL). Owing to the conditions under which samples were collected, CVL were not collected according to the menstrual cycle stage. Since current methods of measuring GTVL do not distinguish between infectious and non-infectious HIV [24], [25], we used the TZM-bl assay to measure HIV infectivity of CVL specimens and correlated these data with GTVL, and PVL in our HIV(+) patient population. We evaluated 32 CVL from HIV infected women for infectious virus. CVL were obtained from HIV(+) women, who had CD4+ cell counts greater than 350 cells/mm^3^ and were not on anti-retroviral (ARV) therapy. Samples were diluted 1∶4 in media (final pH 7.0–7.2) and added to TZM-bl cells to assess whether virus present in CVL was infectious. As shown in Figure 1, CVL from 3 of 32 women contained virus capable of infecting TZM-bl cells. On further analysis, we observed that of the three women, 2 had bacterial vaginosis, a known enhancement factor for HIV infection [31], and 1 had a Trichomonas infection. We found no correlation between GTVL (Table 1), PVL, and infectious virus in CVL. Of the 3 women with infectious virus (# 11, 25, 29), all had MIP3α and HIV specific IgG levels that were lower than average (Table 2).

**Table 2:** Anti-HIV-1 activity and levels of antimicrobials in secretions from HIV(+) patients.

Patient	X4		% Inhibition	R5			(pg/ml)		
Number	IIIB	NL4.3	YU2.c	BaL	CH077.c	HBD2	Elafin	MIP3α	SLPI
1	66	82	12	30	52	50344	10499	1942	108000
2	51	86	−57	26	34	44635	7441	246	103000
3	30	82	53	26	21	2089	4260	21	55340
4	85	84	30	46	37	34056	7820	481	105000
5	62	70	−20	34	27	42438	9746	346	83710
**6**	−60	−46	−86	−58	−43	10	2705	4	18040
7	90	87	1	81	55	48638	3226	669	112000
8	63	47	−10	14	36	19925	2718	9	19510
9	65	68	65	60	89	52713	8316	1173	46800
10	−3	10	14	−20	32	36913	5664	405	76160
**11**	77	84	1	−49	−134	60777	5503	4	14310
**12**	53	−9	−79	−2	−39	48809	7548	4	84320
**13**	11	−20	−45	14	−6	23453	8248	19	87550
14	58	−14	24	52	66	6539	1437	47	79190
15	77	66	−9	56	53	7453	6800	136	32650
16	98	99	35	77	77	42876	1824	55	42400
17	19	23	−19	−8	57	1375	3182	113	22250
18	99	96	66	92	87	46164	32	15	35660
19	100	101	82	98	78	65573	8703	3409	107000
20	100	99	91	90	96	50670	4989	2469	100000
21	93	78	−2	75	47	28611	3206	136	49780
22	79	−7	66	15	41	22319	5328	62	38230
23	89	78	35	67	75	38803	7040	59	55380
24	25	−19	23	0	25	10	226	36	12340
**25**	−23	16	43	−55	69	41898	6465	43	52480
**26**	−61	29	25	−65	−31	26324	4316	146	121000
27	93	99	67	89	88	28985	3368	101	42770
28	95	93	67	63	50	49191	4307	40	27350
**29**	−15	61	64	−32	63	49555	3516	246	51640
30	95	100	92	86	96	15506	2	201	30330
31	38	62	49	31	72	37105	1894	12	8530
32	69	91	35	52	65	55500	4544	446	103000
**MEDIAN**	**66**	**74**	**28**	**33**	**53**	**37954**	**4430**	**107**	**52060**
**INTERQUARTILE RANGE**	**28, 92**	**20, 89**	**−6, 65**	**−1, 71**	**30, 74**	**21122, 49000**	**2950, 7241**	**29, 376**	**31490, 93775**

### CVL from HIV(+) women have anti-HIV activity

**Figure 2:**
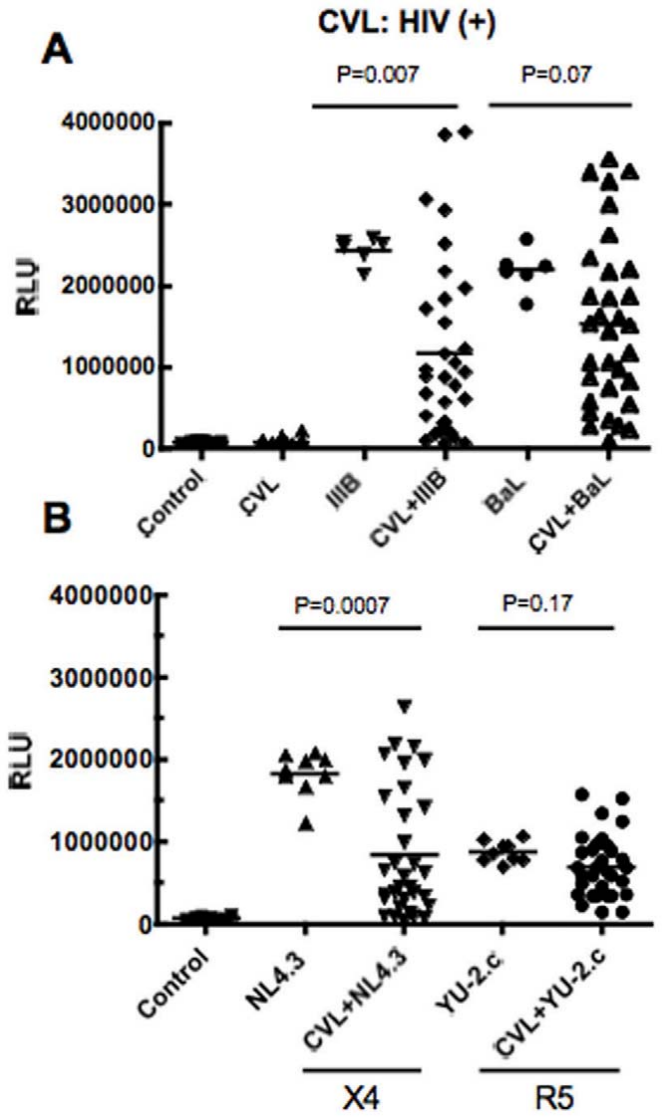
Measurement of antiviral activity in CVL from HIV(+) women. Thirty two HIV(+) CVL were diluted 1∶4 and incubated with HIV-1 IIIB (X4-tropic) and BaL (R5-tropic) (Panel A) or NL4.3 (X4) and YU-2.c (R5) (Panel B) at a multiplicity of infection (MOI) of 1 for 1h at 37°C prior to infecting TZM-bl cells. Data points in each graph represents one individual patient. Media, “Control”, and “CVL only” wells (Panel A) were set up as negative controls, and replica wells of virus (IIIB, BaL, NL4.3 and YU-2.c) were set up as positive controls. Significant differences (p values) between the mean of the control (virus alone) and that of the cognate virus+CVL are shown in the Figure.

To determine whether CVL from HIV(+) women inhibit HIV infection of target cells, CVL were incubated with either X4- or R5- tropic HIV-1 at MOI = 1 for 1h at 37°C prior to the measurement of HIV-1 infectivity via the TZM-bl assay. Among individual subjects, we found a spectrum of anti-HIV activity ranging from −134% to 100% inhibition in CVL (Figure 2). In Table 2, as has been reported by others [32]–[36], we found that some CVL, rather than inhibiting HIV infection, had a stimulatory effect on HIV infectivity without changing background RLU values. Values reported as less than 0% in Table 2 indicate stimulation and are included for completeness. The demonstration of enhancement of viral infection beyond control HIV infection was observed in 33 of 160 assays (20%). The pattern of inhibition was not consistent across viral strains, except in Subject 6 who had below average levels of endogenous microbicides. As seen in Table 2, when compared to virus alone, anti-HIV activity was measurable against both X4 and R5 viruses for the majority of CVL tested. In particular, we found that CVL from some patients had anti-HIV activity between 86–100% across all viruses examined (Patients # 20 and 30). In other cases, we found that anti-HIV activity varied with the virus tested (Patients # 7 and 21). As seen in Table 2, no significant differences were observed among median values for each viral strain examined. Overall, to the best of our knowledge, this is the first demonstration of anti-HIV activity in CVL from North American HIV(+) women who are healthy, and not on ARV therapy.

### CVL from HIV(−) women have anti-HIV activity

**Figure 3:**
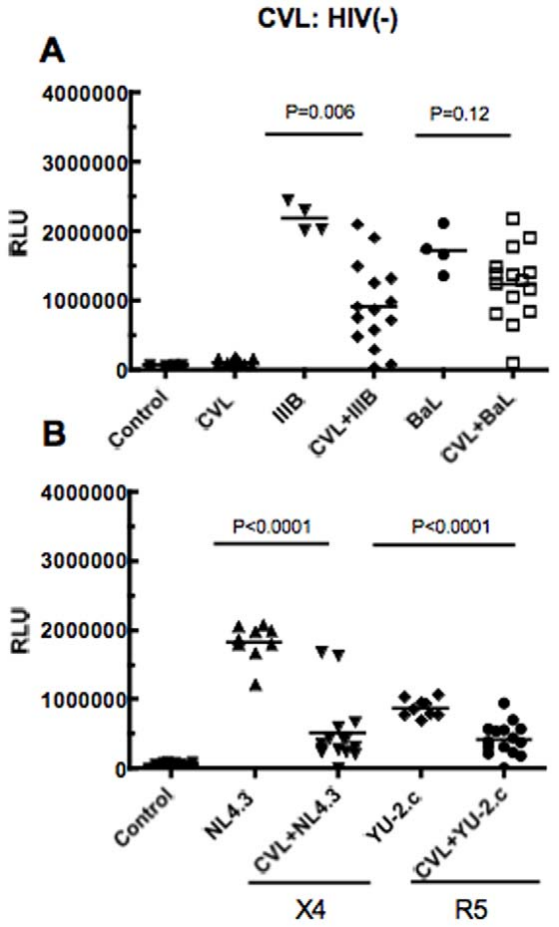
Analysis of antiviral activity in CVL from HIV(−) women. Fifteen HIV(−) CVL were diluted 1∶4 and incubated with HIV-1 IIIB (X4-tropic) and BaL (R5-tropic) (Panel A) or NL4.3 (X4) and YU-2.c (R5) (Panel B) at MOI 1 for 1h at 37°C prior to infecting TZM-bl cells. Media only “Control” and virus only (IIIB, BaL, NL4.3 and YU-2.c) wells were set up as positive controls. Each data point in graph represents one individual patient. Data points in each graph represents one individual patient. Significant differences between control (virus alone) and virus+CVL are indicated in Figure.

To evaluate whether CVL from HIV(−) women had endogenous anti-HIV activity, samples from 15 healthy women were obtained from the RI HER Repository. As shown in Table 3, we found general inhibition of HIV infection of target cells. Similar to HIV(+) women, CVL from HIV(−) women showed limited enhancement of HIV infectivity beyond control infections (3 of 75 assays). Although 5/15 HIV(−) women had BV (# 3,4,5,10,15), we found no correlations of these samples with anti-HIV activity. As seen in Figures 3A and B, we found that some CVL had potent anti-HIV activity while others had little to no activity. As with HIV(+) healthy women, there was a wide diversity of CVL inhibition with individual viruses.

**Table 3:** Anti-HIV-1 activity and levels of antimicrobials in CVL from HIV(−) women.

Patient	X4		% Inhibition	R5			(pg/ml)		
Number	IIIB	NL4.3	YU-2.c	BaL	CH077c	HBD2	Elafin	MIP3α	SLPI
1	14	79	41	29	73	67455	18792	53	75520
2	70	91	39	66	71	20308	15764	76	51880
3	47	85	85	41	82	5487	1892	39	6000
4	66	71	93	26	87	222	4606	26	58520
5	104	97	98	31	96	1780	141	118	19190
6	34	90	44	42	78	3306	6459	124	75810
7	92	94	89	59	96	22288	2327	97	67570
8	69	85	59	22	75	906	2238	448	112000
9	46	10	66	−1	50	19599	14456	90	48170
10	84	93	70	−7	75	1067	3849	155	91070
11	75	77	69	18	79	4	5952	721	45080
12	101	103	105	98	74	115	214	4344	93330
13	61	92	3	59	69	27028	12821	29	51720
14	5	12	40	−27	77	45	6412	71	39960
15	61	71	24	15	64	2394	8647	239	73560
**MEDIAN**	**66**	**85**	**66**	**29**	**75**	**2394**	**5952**	**97**	**58520**
**INTERQUARTILE RANGE**	**46, 84**	**71, 93**	**40, 89**	**15, 59**	**71, 82**	**222, 20308**	**2238, 12821**	**53, 239**	**45080, 75810**

### Antiviral activity in CVL from HIV(+) and HIV(−) women against an infectious molecularly cloned, transmitted/founder virus

**Figure 4:**
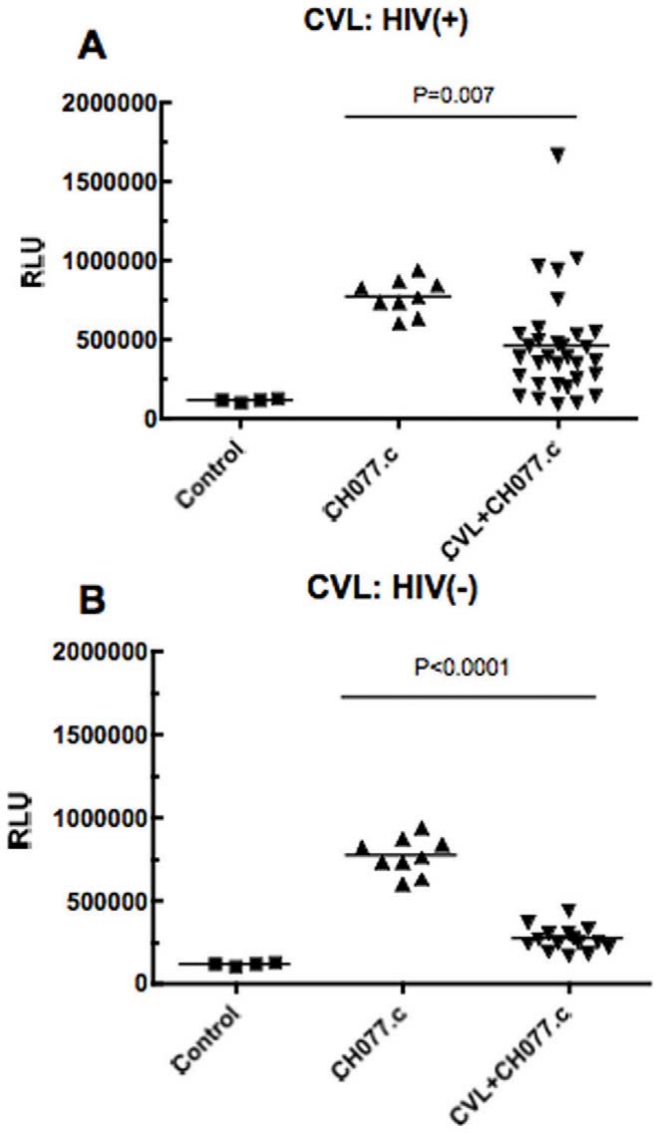
Analysis of CH077.c (R5; transmitted/founder Virus) activity in CVL from HIV(+) and HIV(−) women. Thirty two HIV(+) CVL and 15 HIV(−) CVL were diluted 1∶4 and incubated with the R5 transmitted/founder HIV-1 virus CH077.c at MOI 1 for 1h at 37°C prior to infecting TZM-bl cells. Control wells incubated with CVL alone were set up as negative controls and CH077.c wells were set up as positive controls. Each data point in Figure 4A represents one of 32 individual patients. In Figure 4B each data point represents one of 15 individuals. Significant differences between control (CH077.c) and CH077.c + CVL are indicated in Figure.

Recent studies focusing on initial HIV transmission have identified the genetic sequences of transmitted/founder HIV-1 viruses responsible for the establishment of initial infection [27]. We evaluated the impact of CVL from HIV(+) and HIV(−) women on infectivity of an infectious molecular clone containing the complete nucleotide sequences of transmitted/founder virus, CH077.c (Ochsenbauer *et al.* in preparation). As shown in Figures 4A and 4B, when anti-HIV activity was evaluated between different viruses, we found that transmitted/founder virus (CH077.c) was inhibited by CVL from both healthy HIV(+) and HIV(−) women as were laboratory-adapted viruses (IIIB, NL4.3, BaL, and YU-2.c) shown in Tables 2 and 3. These findings suggest that the CH077.c transmitted/founder virus is as susceptible to inhibition by FRT endogenous microbicides as the other strains tested.

### CVL from HIV(+) and (−) women contain a spectrum of antimicrobials

To define the mechanism(s) through which CVL from HIV(+) and (−) women protect target cells from HIV infection, we analyzed CVL specimens for key endogenous microbicide candidates which we and others have shown in cell culture to have antiviral activity [9]–[12]. Tables 2 and 3 contain a detailed breakdown of anti-HIV activity as well as the concentrations of 4 known anti-HIV molecules measured in individual CVL samples. Analysis of CVL from HIV(+) and (−) women for SLPI, trappin-2/elafin, MIP3**α**, and HBD2 indicated that each of these antivirals are present in CVL irrespective of HIV status. Recognizing that CVL specimens from HIV(+) and (−) women were obtained at different times and from different repositories with slightly different protocols, it was surprising to observe that concentrations of MIP3**α** and SLPI were virtually identical (Tables 2 and 3). Moreover, the concentrations of SLPI in CVL in our studies were comparable to that reported previously by others [15]. Unexpectedly, we found that HBD2 levels were approximately 3-fold greater in HIV(+) women than that seen in HIV(−) women. In contrast, HIV(−) women had significantly (p<0.05) higher values of trappin-2/elafin than HIV(+) women. These data suggest that HIV infection alters the distribution of some endogenous microbicides in FRT secretions.

### Anti-gp160HIV IgA and IgG antibodies in CVL from HIV(+) women

To determine whether the CVL of HIV(+) women had antibodies against HIV, we assessed CVL samples for the presence of anti-HIV gp160 specific IgG and IgA antibodies using 15 mOD as a cut-off for detection [29]. As seen in Table 4, we found that 30 out of 32 CVL from HIV(+) women had anti-gp160HIV IgG antibodies, while none were positive for anti-HIV IgA antibodies (data not shown). Neither anti-HIV gp160 IgG nor IgA antibodies were detected in CVL from HIV(−) women (Table 4).

**Table 4:** HIV GP160-specific IgG in CVL from HIV(+) and HIV(−) women.

Patient #	32 HIV+ Patient ID	IgG	15 HIV- Patient ID	IgG
1	B071c	583	40048	1.7
2	B083c	907	400993	1.7
3	B037c	148	400415	1.7
4	B057c	645	402382	1.5
5	B060a	>1000	402757	1.2
6	B059c	13	401687	1.8
7	B013c	>1000	400868	1.4
8	B006c	37	401927	1.2
9	B078c	1106	400937	0.7
10	B062a	745	401541	1
11	B011a	272	401701	1.5
12	B027c	1190	400802	12.6
13	B074c	29	400824	2.2
14	B061c	43	402428	1.6
15	B079c	529	400982	0.4
16	B015a	562		
17	B086d/a	107		
18	B073b	532		
19	B068c	>1000		
20	B018c	>1000		
21	B012c/a	233		
22	B067c	26		
23	B077d/a	95		
24	B051d/c	2.7		
25	B085c/a	25		
26	B017c	54		
27	B029c	914		
28	B048c	134		
29	B049c	128		
30	B047c	534		
31	B058c/a	64		
32	B075c	501		

The results are in milliOD per minute but correspond directly to the amount of binding antibody. 15 is the usual cut-off by this criteria, only two HIV infected person lacked antibody. No antibodies were detected in HIV(−) CVL.

### Anti-HIV activity correlates with specific antimicrobials and anti-HIV IgG in CVL from HIV(+) women

Table 5 summarizes the correlation by Spearman analysis between % inhibition of HIV infection of target cells by each strain of HIV with concentrations of the four antimicrobials (HBD2, trappin-2/elafin, MIP3α, SLPI) and anti-HIV gp160 IgG. Of the 4 antimicrobials tested, we found positive correlations between the levels of HBD2 and MIP3α as well as anti-HIV IgG with inhibition of some HIV strains. In particular, MIP3α correlated with anti-HIV activity against all 3 of the R5 viruses but neither of the X4 viruses. In contrast, we found no correlation of anti-HIV activity with SLPI and trappin-2/elafin. Similarly, when we analyzed HBD2, MIP3α trappin-2/elafin, and SLPI vs anti-HIV activity in CVL from HIV(−) women, we found no evidence of a positive correlation (data not shown).

**Tables 5:** Correlation of levels of antimicrobials and anti-HIV IgG in CVL with anti-HIV activity against 5 different viruses.

		% Reduction in Target Cell Infection with Indicated Strain				
HIV+ (N = 32)	IIIB (X4)	NL4.3 (X4)	BaL (R5)	YU-2.c (R5)	CH077.c (R5)	Percent reduction against all viruses, summed quartiles
HBD2						
Spearman's ρ	0.391	0.28	0.53	0.288	0.283	**0.395**
P value	**0.027**	0.121	**0.002**	0.109	0.116	**0.025**
Elafin						
Spearman's ρ	−0.053	−0.178	−0.009	−0.095	−0.198	**−0.091**
P value	0.772	0.331	0.961	0.605	0.277	**0.619**
MIP3a						
Spearman's ρ	0.278	0.271	0.425	0.382	0.4	**0.397**
P value	0.124	0.134	**0.015**	**0.031**	**0.023**	**0.025**
SLPI						
Spearman's ρ	0.033	−0.053	0.184	0.136	−0.017	**0.079**
P value	0.859	0.775	0.315	0.46	0.925	**0.668**
Anti-HIV IgG						
Spearman's ρ	0.484	0.117	0.595	0.517	0.283	**0.502**
P value	**0.005**	0.526	**0.001**	**0.002**	0.117	**0.003**

To further assess the overall contribution of each antimicrobial and anti-HIV IgG for protection against multiple HIV viral strains, the percent reduction in HIV infection of TZM-bl cells for each isolate was divided into quartiles, and a score assigned to each quartile. Then we assessed the correlation of levels of each antimicrobial or anti-HIV IgG with the summed quartile scores using Spearman correlation coefficients as above. As seen in Table 5 (right column), we found significant correlations, identical to that seen above with Spearman analysis, between the levels of HBD2, MIP3α and anti-HIV IgG with the summed percent reduction in HIV infection. These data are further supportive evidence of the importance of HBD2, MIP3α and anti-HIV IgG in protecting the FRT from multiple strains of HIV.

## Discussion

The present study evaluated the presence of anti-HIV activity in CVL from HIV(+) healthy and HIV(−) women. When analyzed by ELISA, CVL from HIV(+) and HIV(−) women were found to contain a spectrum of endogenous microbicides with activity against both X4- and R5-tropic HIV.

When CXCR4 and CCR5 tropic HIV-1 were incubated with CVL from HIV(+) women prior to addition to TZM-bl cells, anti-HIV activity in CVL ranged from none to 100% inhibition with some showing enhancement, depending on the viral strains used. CVL from HIV(−) controls showed comparable anti-HIV activity. Importantly, CVL from HIV(+) and HIV(−) women demonstrated potent antiviral activity against a molecular clone of a transmitted/founder virus, CH077.c, that was comparable to laboratory strains. Measurement of CVL for antimicrobials demonstrated that HBD2 and MIP3α correlated with anti-HIV activity as did anti-gp160 HIV IgG antibodies in CVL from HIV(+) women.

The present studies demonstrate that CVL from HIV(+) and HIV(−) women contain at least four microbicides with known anti-HIV activity. We and others have examined SLPI, trappin-2/elafin, MIP3α, and HBD2 and found that each has anti-HIV activity [13], [14], [37]–[39]. Unexpectedly, in the present study, we found that anti-HIV-1 activity in CVL from HIV(+) women correlated with CVL levels of MIP3α and HBD2 but not with SLPI or trappin-2/elafin. Interestingly, MIP3α concentrations in HIV(+) CVL positively correlated with anti-HIV activity against all 3 R5 HIV strains, including the transmitted/founder virus CH077.c. In addition, anti-HIV activity in CVL from HIV(+) women correlated significantly with anti-gp160HIV IgG antibodies. This is the first study to correlate anti-HIV activity of healthy HIV(+) CVL from North American women with the levels of anti-HIV antibodies. While we have not established that these antibodies possess neutralizing ability, these data clearly suggest a role for antibodies in the immune defense against HIV transmission. Taken together, these findings indicate that CVL from HIV(+) and (−) women contain endogenously produced antimicrobials that inhibit HIV infection, and in doing so may limit both acquisition and transmission of infection.

An unexpected finding was that approximately 3/32 samples from HIV(+) CVL examined contained virus capable of infection, and that infectious virus was independent of plasma and genital tract viral load. While very few studies have measured infectious virus in CVL, others have reported similar low numbers of women with CVL infectious virus [24]. In our study, the 3 women with infectious virus had MIP3α and HIV-specific IgG levels that were lower than average (Table 2 and Table 4). This finding suggests that endogenous antimicrobials such as MIP3α and HIV-specific IgG antibodies are too low to inactivate virus. These findings are of particular importance because they suggest that local protection in CVL may inactivate infectious virus to limit sexual transmission. Others have demonstrated that low vaginal pH can inactivate HIV [40]. That this is unlikely is suggested from studies in which vaginal pH was measured both prior to and immediately following ejaculation. In all cases, vaginal pH was neutralized from 5.6 to 7.2 within 6 seconds [41], [42]. These findings suggest that antivirals, such as MIP3α measured in the present study along with other endogenous microbicides, are responsible for viral inactivation. Further studies are essential to identify the molecules involved in limiting the presence of infectious virus in genital tract secretions.

It is well established that during sexual transmission, R5 strains of HIV are selectively transmitted over X4 strains, although the mechanisms for this are poorly understood [43]–[45]. An unexpected finding in the present study was the variation in antiviral activity in CVL specimens between individuals and against different viruses. This range of inhibition existed between viruses of the same tropism (X4: IIIB and NL4.3; R5: BaL, YU-2.c and CH077.c) and was independent of subject demographic characteristics. One explanation is that variations in viral envelopes might lead to differences in sensitivity to antivirals in CVL. For example, SLPI and defensins have been shown to inhibit HIV infection based on tropism [46], [47]. Alternatively, since CVL contain a spectrum of antimicrobials that vary with stage of the menstrual cycle [15], it is likely that variations in both the quality and quantity of antimicrobials between individuals contribute to the diversity of antiviral activity seen in this study. The observation that MIP3α, which we have shown to inhibit HIV infection [13], correlated positively with anti-HIV activity against the R5, but not the X4, HIV strains suggests a unique protective role for this antimicrobial against HIV-1 infection. Whether viral differences, cycle stage or heterogeneity of antimicrobials in CVL samples are responsible remains to be determined.

Anti-HIV activity of CVL from HIV(−) women did not correlate with the levels of any of the measured microbicides (data not shown). One explanation for this finding is that the number of HIV(−) CVL samples analyzed in this study [15] was too low. An alternative explanation is that the anti-HIV activity of CVL stems from a combination of multiple endogenous microbicides and that the assessment of no single factor adequately captures the sum total anti-HIV activity of any one woman's CVL microbicides [15], [16], [48]. For example, others have shown that CVL contain the cathelicidin peptide LL37, calprotectin, alpha defensins and lactoferrin, each of which has limited innate antimicrobial activity but can act in synergy to inhibit HIV [9], [49]. What appears not to be involved is pH. Although low pH can selectively destabilize the viral envelope leading to altered viral infectivity [24], all samples tested in this study were diluted in buffered media and found to be pH neutral (pH 7–7.2) prior to assay for anti-HIV activity.

Our studies demonstrate that CVL from a subset of women enhanced HIV infection of target cells. This suggests that CVL from HIV(+) women may contain factors that enhance HIV infection. Such molecules include proinflammatory cytokines such as IL-6, IL-8, TNFα, and IL-1â. These are often present in the CVL in association with pre-existing infections with STI such as bacterial vaginosis (BV), HSV-2, *Trichomonas vaginalis*, *Neisseria gonorrhea*, and *Candida albicans* [50]–[52]. In fact, BV infection has been frequently found to be a major correlate for enhanced HIV replication in the FRT presumably through the enhanced production of proinflammatory cytokines. Several of these factors can directly enhance HIV replication by stimulating the HIV LTR [32]–[34], [52], [53]. Another molecule found in serum and mucosal secretions and associated with enhanced HIV replication is myeloid related proteins MRP 8/14 [35]. Another protein, a scavenger receptor gp340, has been shown to be expressed by cervical and vaginal epithelium and promote trans-infection of HIV even when the epithelium remains intact [36], [54].

Some recent studies have demonstrated that the mere presence of anti-HIV molecules in the genital tract does not necessarily correlate with HIV neutralization activity in vivo [55], [56]. This is because some of these anti-HIV molecules such as RANTES, LL37 and MIP3a have potent chemotactic activity and can attract target cells to the site of infection thereby causing an enhancement effect. Another factor that might explain the variability in HIV neutralization ability of CVL is the amount of bioactive anti-HIV molecules present at a given time. Bioactivity is often determined by the presence of multiple families of proteases in the genital tract that are responsible for specific activation and deactivation of immune factors. The Cathepsin family of proteases regulates the family of matrix metalloproteases, which are themselves responsible for activating/deactivating innate immune factors including the anti-HIV molecules SDF-1 and HNP1 [57]–[60]. Cathepsins are also responsible for directly regulating anti-HIV innate factors [61]–[63]. For example, Cathepsin D, a cysteine protease present in vaginal secretions [49] has been shown to enhance HIV replication [64], [65]. Although the mechanisms are unclear, it is known that Cathepsin D inhibits MIP3α [61], a known anti-HIV factor in CVL [13]. Kallikreins (KLK) are another family of serine proteases present in the genital mucosa that can activate/deactivate multiple immune factors in the FRT [66] including LL37, a potent anti-HIV molecule [67], [68]. Finally, CD26/dipeptidyl peptidase IV (DPIV) is a serine protease responsible for the cleavage and inactivation of chemokines such as RANTES and SDF-1, which are involved in blocking HIV entry [69], [70]. A further level of complexity arises in that, beyond their ability to activate and inactivate FRT antimicrobials, these protease families are regulated throughout the menstrual cycle by protease inhibitors present in the genital secretions. Several protease inhibitors such as SLPI and trappin-2/elafin are also known anti-HIV molecules [12], [37], [71].

We examined the role of specific endogenous microbicides in the reduction of HIV infection of target cells by CVL from HIV(+) and HIV(−) women, finding that the levels of HBD2 and MIP3α correlated significantly with inhibition of infection. HBD2 is reported to inhibit infection through direct interaction with the virus, as well as decreasing expression of CXCR4, the co-receptor for X4 HIV-1 viruses (but not CCR5) in peripheral blood mononuclear cells and T lymphocytic cells as shown by confocal microscopy and flow cytometry [46]. Sun et. al. did not find a decrease in co-receptor expression with HBD2 treatment, and suggest an effect on the intracellular environment that inhibits HIV [72]. We found that HBD2 and SLPI are present in CVL at concentrations comparable to previous reports [15], [73], [74]. Notably, CVL from HIV(+) women had significantly higher HBD2 than HIV(−) women, suggesting that HIV infection upregulates the production of this potentially protective endogenous microbicide.

One explanation for the variability seen in anti-HIV activity (innate and specific IgG antibodies) in the present study is that it was not possible to collect CVL from HIV(−) and HIV(+) women according to stage of the menstrual cycle. As discussed elsewhere [48], innate and adaptive immunity throughout the female reproductive tract are under hormonal control. For example, we found that midcycle suppression of the humoral immunity by estradiol, which confirms the findings of other laboratories [75], [76], extends to endogenous antimicrobials in CVL [15]. Analysis of the concentrations of cytokines, chemokines and antimicrobials in CVL indicated that SLPI, HBD2, HNP1-3 and lactoferrin dropped significantly at midcycle (day 13) and remained depressed for 7–10 days prior to returning to proliferative phase levels just prior to menstruation. Therefore, owing to hormonal changes during the menstrual cycle, antimicrobials and antibodies may not be present at concentrations sufficient to exert anti-HIV effects. Thus, without compensating mechanisms, innate immune protection is suppressed transiently at midcycle to optimize the chances for successful fertilization, implantation and pregnancy. What is likely is that antiviral activity in CVL is the net and possibly synergistic result of 12–20 antimicrobials present at varying concentrations over the course of the menstrual cycle. Studies to assess the spectrum of antimicrobials in CVL and the ways in which each is altered during the menstrual cycle are essential for a complete understanding of the role of the innate immune system in protection and control of sexual transmission of HIV.

Other studies have demonstrated the presence of anti-HIV antibodies in CVL from HIV(+) women [1], [77]–[79], but to the best of our knowledge, ours is the first to correlate the levels of these antibodies with anti-HIV activity in CVL from healthy North American HIV(+) women not on any ARVs. These results should prompt an assessment of whether these antibodies exhibit neutralizing activity against HIV, or if the correlation with protection of target cells arises from other mechanisms. Binding but non-neutralizing IgG in the CVL of HIV(+) women has been shown to be protective by lowering viral loads and presenting better clinical outcomes [80]. Such antibodies are believed to protect by inducing ADCC (antibody dependent cellular cytotoxicity) and ADCVI (Antibody dependent cell mediated viral inhibition) [81]. In fact, the recent vaccine trial in Thailand shows preliminary data indicative of protective effects from binding but non-neutralizing antibodies [82]. We also found, similar to other studies [78], [79], [83], that CVL from HIV(+) women lacked anti-HIV specific IgA antibodies, suggesting that IgA in the FRT might not play a substantive role in protection from HIV transmission.

In summary, this study demonstrates that CVL from healthy HIV(+) and HIV(−) women have intrinsic anti-HIV activity and that this activity is most likely mediated through a spectrum of endogenously produced antimicrobials which are capable of inhibiting X4 and R5 viruses. Whereas a spectrum of factors capable of mediating antimicrobial protection are present in CVL, the levels of HBD2, MIP3α and HIV specific IgG antibodies correlated with protection of target cells from infection with HIV. These findings highlight the need for additional studies to more fully understand the influences of the innate immune system and its regulation by sex hormones during the menstrual cycle, pregnancy and following menopause in immune protection throughout the reproductive tract. Moreover, it suggests that a clear understanding of innate protection in the female reproductive tract may lead to new candidate microbicides and approaches for microbicide-mediated immune protection.
